# Case Report: PD-L1-negative advanced bladder cancer effectively treated with anlotinib and tislelizumab: A report of two cases

**DOI:** 10.3389/fonc.2023.1164368

**Published:** 2023-04-14

**Authors:** Teng Li, Wuyun Hu, Lan Jin, Xianghua Yin, Dongxu Kang, Longzhen Piao

**Affiliations:** Department of Oncology, The Affiliated Hospital of Yanbian University, Yanji, Jilin, China

**Keywords:** urothelial cancer (UC), anlotinib, fibroblast growth factor receptor (FGFR), tislelizumab, PD-L1

## Abstract

Second-line treatment for metastatic or locally advanced urothelial cancer (UC) is limited. Immunotherapy is approved as a second-line treatment for metastatic UC. Its use as a first-line agent is limited to patients who are ineligible for cisplatin-based treatments. The fibroblast growth factor receptor (FGFR) inhibitor, erdafitinib, can be applied as a third-line approach after the failure of these prior treatments in eligible patients. Therefore, it is especially important to combine limited drugs for second-line treatment of advanced or metastatic UC. Anlotinib is a multiple tyrosine kinase inhibitor agent with both anti-angiogenic and FGFR inhibitory effects. For two patients with advanced and metastatic UC, we combined anlotinib and tislelizumab therapy even though there is no indication of its use. We describe two patients with programmed death ligand-1 (PD-L1)-negative advanced bladder cancer, one with FGFR3 mutation and another with FGFR3 wild type. Both patients had progressed after first-line chemotherapy with gemcitabine and cisplatin. We selected anlotinib in combination with tislelizumab, a programmed death-1 (PD-1) immune checkpoint inhibitor, for second-line treatment. Responses were evaluated as partial remission in both cases, who achieved up to 12 months of progression-free survival with no significant adverse events. Two patients with PD-L1-negative UC underwent second-line therapy using tislelizumab in combination with anlotinib, and the efficacy was better than that of tislelizumab alone. These results suggest that anlotinib may act synergistically with tislelizumab in the treatment of UC.

## 1 Introduction

First-line therapy for metastatic or locally advanced urothelial cancer (UC) includes cisplatin-based chemotherapy combinations, with platinum-based regimens resulting in median overall survival of just over 1 year (1, 2). Although considered a chemo-sensitive disease, most patients with metastatic or locally advanced UC relapse after cisplatin-based first-line treatment. None of the drugs currently commonly used (i.e., paclitaxel, carboplatin, and/or pemetrexed) are approved by the Food and Drug Administration (FDA) for second-line systemic treatment (3). The immune checkpoint inhibitor (ICI) class of agents is active for the second line and beyond the treatment of UC post-platinum progression. More recently, ICIs, including several anti-PD-L1 and anti-PD-1 antibodies, have shown promising activity in first-line and post-platinum settings (4–6). In patients with advanced urothelial carcinoma who progressed following platinum-based chemotherapy, Bellmunt et al. (7) found that pembrolizumab only increased overall survival by 3 months when compared to second-line paclitaxel, docetaxel, or vinflunine, independent of tumor PD-L1 expression status (7).

Given the extensive functions of fibroblast growth factor/fibroblast growth factor receptor (FGFR) signaling, dysregulation of this pathway has both oncogenic and tumor-suppressive potential (8), and dysregulated FGFR signaling is associated with tumor proliferation, survival, migration, invasion, and angiogenesis (9). Approximately 15%–20% of patients with advanced or metastatic UC have FGFR alterations, and a new generation of FGFR-tyrosine kinase inhibitors (TKIs), including erdafitinib, AZD4547, and rogaratinib, has been proposed (10). The BLC2001 phase II trial enrolled 99 patients with UC with FGFR2/FGFR3 alterations and reported an objective response rate (ORR) to erdafitinib of 40%, with median progression-free survival (PFS) of 5.5 months and median overall survival (OS) 13.8 months. FDA approval of erdafitinib has provided a new treatment option for patients with FGFR-altered UC who progress after platinum-based chemotherapy.

Angiogenesis is a fundamental process during UC initiation and progression (11). Studies on bevacizumab, pazopanib, and ramucirumab have shown improved response rates when these agents were added to chemotherapy in selected patients, but they did not confer OS benefits in randomized controlled studies (12).

In advanced or metastatic UC, second-line treatments, such as some chemotherapeutic agents, ICIs, FGFR inhibitors, and anti-angiogenesis therapy, have achieved a level of efficacy but remain unsatisfactory. Therefore, future studies should evaluate how specific sequences and combinations of treatment contribute to survival benefits. ICIs have achieved remarkable clinical success, yet their efficacy in “immunologically cold” tumors has been modest; therefore, how to turn “cold” into “hot” tumors, to improve immunotherapy efficacy, has become a research focus.

Anlotinib is an anti-angiogenic, orally administered TKI drug that targets vascular endothelial growth factor receptor (VEGFR), FGFR, platelet-derived growth factor receptor (PDGFR), and c-Kit (13). Here, we report two patients with advanced bladder cancer, both of whom were PD-L1-negative, who were treated with anlotinib combined with tislelizumab. Both patients achieved up to 12 months of PFS with no significant adverse events.

## 2 Case presentation

### 2.1 Case 1

**Figure 1:**
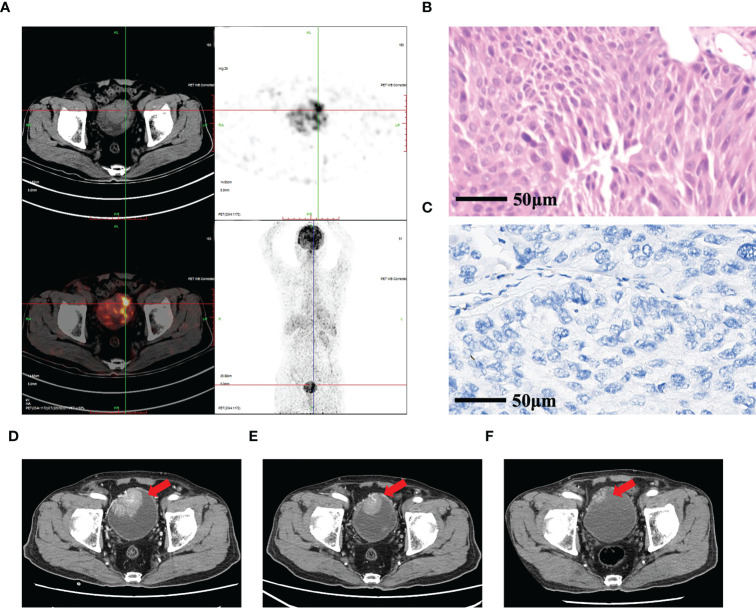
Pathological and imaging examination of case 1. **(A)** Whole-body PET/CT showing bladder thickening, multiple nodular and mass-like soft tissue shadows in the cavity, and increased metabolism. **(B)** Pathological results of hematoxylin and eosin cytology staining of a tumor biopsy specimen. **(C)** PD-L1 expression was evaluated by immunohistochemistry. **(D)** Enhanced CT of the abdomen before treatment showing a 5.3 × 4.0 cm mass in the bladder with significant enhancement in the arterial phase (red arrow). **(E)** After two cycles of treatment, enhanced CT of the abdomen revealed that the tumor shrank to 4.1 × 3.2 cm (red arrow). **(F)** After 14 cycles of treatment, enhanced CT of the abdomen revealed that the size of the tumor was 1.2 × 1.0 cm (red arrow).

A 77-year-old man with a 30-year history of smoking 10 cigarettes per day presented with painless gross hematuria in the fall of 2013 and was ultimately diagnosed with muscle-invasive bladder cancer in a Korean hospital. Transurethral resection of bladder tumor (TURBT) was conducted, followed by six cycles of first-line treatment (gemcitabine and cisplatin). On April 3, 2021, the patient was admitted to the Affiliated Hospital of Yanbian University due to hematuria. Physical examination did not reveal any abnormalities. Given the possible progression of the patient’s condition, we conducted a positron emission tomography (PET)/computed tomography (CT) examination directly. The result showed bladder thickening, multiple nodular and mass-like soft tissue shadows in the cavity, and increased metabolism; there was no evidence of distant metastasis (**Figure 1A**). The patient also underwent cystoscopy tumor biopsy, pathological examination, and PD-L1 expression detection. Summary immunohistochemistry results were as follows: CK20(+), CK7(+), P53(−), and Ki-67 (upshifted positive cells). The results of hematoxylin and eosin staining are shown in **Figure 1B**, and the results of PD-L1 detection are shown in **Figure 1C**. PD-L1 was detected using the VENTANA PD-L1 (SP263) antibody, and patients are considered PD-L1-negative if involved tumor-infiltrating immune cells (ICs) were <1%. This patient also underwent next-generation sequencing (NGS) and was found to have an FGFR3 exon 9 mutation, encoding a Y373C substitution. In summary, the patient was diagnosed with muscle-invasive bladder cancer (rT2N0M0, PD-L1-negative, FGFR3-mutated). Total cystectomy was recommended after a multidisciplinary team discussion but was rejected by the patient.

The patient was treated for two cycles with anlotinib (10 mg, d1–14) and tislelizumab (200 mg, three times weekly). On June 2, 2021, the patient was re-assessed by enhanced abdominal CT, and tumor size decreased from 5.3 × 4.0 cm (**Figure 1D**) to 4.1 × 3.2 cm (**Figure 1E**). Subsequently, the patient continued to receive anlotinib combined with tislelizumab for 14 cycles. On March 31, 2022, the patient underwent contrast-enhanced CT, which showed that the right anterior bladder mass was smaller than the previous (approximately 1.2 × 1.0 cm) (**Figure 1F**). His response was evaluated as partial remission (PR) according to the Response Evaluation Criteria in Solid Tumors (RECIST; version 1.1), and the current regimen is maintenance therapy. PFS has been >12 months.

### 2.2 Case 2

**Figure 2:**
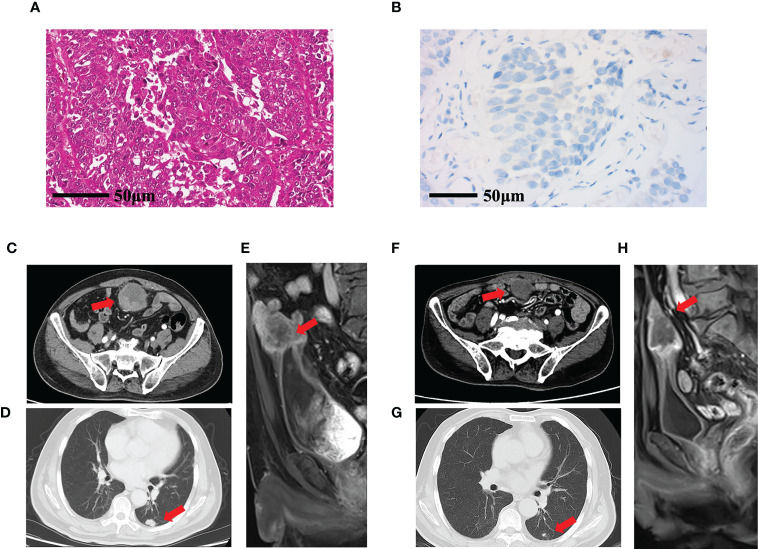
Pathological and imaging examination of case 2. **(A)** Pathological results of hematoxylin and eosin cytology staining of a tumor biopsy. **(B)** PD-L1 expression evaluated by immunohistochemistry. **(C)** Abdominal enhanced CT showing a round nodule measuring 5.4 × 5.5 cm with clear boundaries at the top of the bladder (red arrow). **(D)** Chest CT showing a 1.7-cm nodule in the inferior lobe of the right lung (red arrow). **(E)** Pelvic MRI sagittal axis T2WI showing that the tumor at the base of the bladder was 6.5 × 5.5 cm, with a moderately high signal shadow. **(F)** After 16 cycles of treatment, enhanced CT of the abdomen revealed that the tumor was 3.8 × 3.7 cm (red arrow); **(G)** Chest CT showed that the size of the mass in the lower lobe of the right lung was reduced to 0.8 cm **(H)** Pelvic MRI, sagittal axis, and T2WI showed that the tumor in the bladder base was 5.0 × 3.8 cm, with a moderately high signal shadow (red arrow). T2WI, T2-weighted imaging.

The patient was an 84-year-old man with a history of hypertension and diabetes. One year ago, the patient was diagnosed with muscle-invasive bladder cancer at a local hospital due to painless hematuria and underwent TURBT, followed by one cycle of first-line therapy (gemcitabine and cisplatin). The patient ceased chemotherapy due to adverse reactions to the drugs. On April 2, 2021, he presented at our hospital with a recurrence of painless hematuria, and a biopsy confirmed bladder cancer recurrence (**Figure 2A**). Immunohistochemistry was used to assess the expression of PD-L1 as well (**Figure 2B**), and wild-type FGFR3 was discovered by NGS. The patient underwent abdominal enhanced CT (**Figure 2C**), chest CT (**Figure 2D**), and pelvic magnetic resonance imaging (MRI) (Figure F), which revealed several oval nodules of varying sizes and a maximum diameter of 1.7 cm in both lungs, as well as a round soft tissue density shadow (5.4 × 6.5 × 5.5 cm) toward the base of the bladder. The final diagnosis was stage IV bladder UC with multiple lung metastases.

The patient underwent two cycles of treatment with anlotinib (10 mg, d1–14) combined with tislelizumab (200 mg, three times per week). On May 18, 2021, a pelvic MRI revealed that the patient’s tumor on top of the bladder was 5.0 × 4.2 × 5.0 cm. After two cycles of anlotinib and tislelizumab treatment, efficacy was evaluated as stable disease. The patient continued to receive anlotinib in combination with tislelizumab, during which abdominal enhanced CT and chest CT showed gradual tumor shrinkage. The patient was admitted to our hospital and received abdominal enhanced CT (**Figure 2F**), chest enhanced CT (**Figure 2G**), and pelvic MRI (**Figure 2H**) during the most recent checkup on May 21, 2022. The results revealed that the mass at the top of the bladder had shrunk by 3.8 cm with uneven enhancement. Multiple nodules in both lungs were smaller and less dense than the previous, with a maximum diameter of approximately 0.8 cm. According to RECIST 1.1, the overall outcome rating was PR. Subsequently, the patient developed acute cerebral infarction, failed to continue maintenance treatment, and was lost to follow-up.

## 3 Discussion

Current guidelines for the treatment of advanced UC suggest combination chemotherapy as the first-line treatment, with immunotherapy indicated for patients who relapse after chemotherapy or who are cisplatin-ineligible, with tumors expressing PD-L1. The FGFR inhibitor, erdafitinib, is recommended in the third-line setting after the failure of these prior treatments in eligible patients (14).

The use of ICIs for the treatment of metastatic UC has rapidly increased over recent years. An open-label, randomized, phase III trial compared pembrolizumab versus chemotherapy (paclitaxel, docetaxel, or vinflunine) in 542 patients with advanced UC that recurred or progressed after platinum-based chemotherapy. Data from this trial showed a longer median OS for patients treated with pembrolizumab compared with chemotherapy (10.3 *vs.* 7.4 months; p = 0.002) (7). Two ICIs, durvalumab and atezolizumab, were previously FDA-indicated and recommended in the National Comprehensive Cancer Network (NCCN) Guidelines for the treatment of patients with previously treated advanced or metastatic UC (14). Tislelizumab is a PD-1 inhibitor that has been used to treat locally advanced or metastatic UC with high PD-L1 expression; the ORR of tislelizumab monotherapy for UC was 24%, and the median PFS was 2.1 months (15). Although ICIs have shown efficacy in advanced or metastatic UC, they are influenced by PD-L1 expression level and only achieved 2.9 months longer survival relative to conventional chemotherapy (7). However, our two PD-L1-negative patients who received tislelizumab in combination with anlotinib both achieved PFS > 12 months.

Given that both anti-angiogenesis and immune checkpoint blockade therapies target the tumor microenvironment, a combination of both types of agents may result in a synergistic antitumor effect (12, 16). When combined with tislelizumab, the novel anti-angiogenic TKI anlotinib, which targets VEGFR, FGFR, PDGFR, and c-Kit, demonstrated significant clinical efficacy in our PD-L1-negative cases. This result suggests that anlotinib may directly reduce tumor development and metastasis by changing the microenvironment around the tumor from an immunosuppressive to an immune-permissive state.

Dysregulation of FGFRs has been implicated in various human malignancies, including UC, in which genetic alterations of FGFR1–3 have been implicated (17). Recent research on molecular subtypes and ICIs therapy has shown that UC of the bladder with FGFR3 mutation or high expression has a comparatively low immune signature and lower expression of PD-L1 (18, 19). Inhibition of FGFR3 in FGFR3-activated bladder cancer has been shown to increase PD-L1 protein levels by changing its ubiquitination, which in turn regulates CD8+ T cells from engaging in antitumor activity (20). NEDD4, an E3 ubiquitin ligase of the NEDD4 family of proteins, functions in substrate recognition and attachment of ubiquitin to substrates (21). In UC, it may be phosphorylated by FGFR3 activation and served as a regulator of PD-L1 ubiquitination to regulate CD8+ T cell-mediated immune surveillance (22). Multiple clinical trials are ongoing to evaluate the role of FGFR-targeted therapy, including erdafitinib, the first FDA-approved targeted therapy, in the treatment of UC (23). Loriot et al. (ClinicalTrials.gov number: NCT02365597) reported that the use of erdafitinib was associated with tumor response in 40% of patients who had locally advanced and unresectable or metastatic urothelial carcinoma with FGFR alterations (24). Erdafitinib and other FGFR-TKIs require dysregulated FGFR signaling, such as activation of downstream signal transduction pathways, which can occur in response to FGFR gene alterations, or increased FGF, leading to autocrine and paracrine signaling, and epithelial–mesenchymal transition.

Anlotinib with tislelizumab therapy was used to treat the first case in this study, which was PD-L1 negative with an FGFR3 mutation. This prolonged PFS is above what is generally seen with FGFR-TKI or PD-1 agent therapy provided alone. Although anlotinib is not a specific FGFR3 inhibitor, it can inhibit members of the FGFR family of proteins and has anti-angiogenic characteristics. When combined with tislelizumab, it appeared to have synergistic effects, which may account for the patient’s PFS of >12 months.

There are yet no specific FGFR-3 inhibitors approved in China. Therefore, we combined a PD-1 agent indicated for second-line treatment of advanced or metastatic bladder cancer with the multiple TKI agent anlotinib, which can inhibit the FGF-FGFR signaling pathway, regulate the tumor vasculature, and achieve significant clinical efficacy. Nevertheless, the optimal timing and sequence regimen of a combination of anlotinib and tislelizumab is unclear, and the optimal dose of each agent remains unknown. Finally, the lack of sensitive and efficient predictive biomarkers for anti-angiogenic agents in combination with ICIs impeded the adjustment of the regimen.

Given the limited data on combination treatment with anlotinib and tislelizumab for advanced or metastatic UC to date, this study provides a basis for further development of this approach for second-line UC therapy. We are currently preparing a phase II clinical study of second-line combination treatment with tislelizumab and anlotinib for advanced UC (http://www.chictr.org.cn, Chinese Clinical Trial Register number: ChiCTR2100051595).

## Data availability statement

The raw data supporting the conclusions of this article will be made available by the authors, without undue reservation.

## Ethics statement

The studies involving human participants were reviewed and approved by Medical ethics committee/Affiliated Hospital of Yanbian University. The patients/participants provided their written informed consent to participate in this study. Written informed consent was obtained from participants for the publication of this case report and for the publication of and any identifiable material contained in this article.

## Author contributions

TL and WH carried out the studies. All authors contributed to the article and approved the submitted version.
